# Disease burden of prostate cancer from 2014 to 2019 in the United States: estimation from the Global Burden of Disease Study 2019 and Medical Expenditure Panel Survey

**DOI:** 10.4178/epih.e2023038

**Published:** 2023-03-21

**Authors:** Shen Lin, Dong Lin, Yiyuan Li, Lixian Zhong, Wei Zhou, Yajing Wu, Chen Xie, Shaohong Luo, Xiaoting Huang, Xiongwei Xu, Xiuhua Weng

**Affiliations:** 1 Department of Pharmacy, The First Affiliated Hospital of Fujian Medical University, Fuzhou, China; 2 Department of Pharmacy, National Regional Medical Center, Binhai Campus of The First Affiliated Hospital, Fujian Medical University, Fuzhou, China; 3 Department of Pharmacy, Shishi General Hospital, Quanzhou, China; 4 College of Pharmacy, Texas A&M University, College Station, TX, USA; 5 Journal Center of The First Affiliated Hospital of Fujian Medical University, Fuzhou, China

**Keywords:** Prostate cancer, Global Burden of Disease Study, Medical Expenditure Panel Survey, Disease burden, Healthcare costs

## Abstract

**OBJECTIVES:**

The aim of this study was to evaluate the disease burden of prostate cancer (PC) and assess key influencing factors associated with the disease expenditures of PC in the United States.

**METHODS:**

The total deaths, incidence, prevalence, and disability-adjusted life-years of PC were obtained from the Global Burden of Disease Study 2019. The Medical Expenditure Panel Survey was used to estimate healthcare expenditures and productivity loss and to investigate patterns of payment and use of healthcare resources in the United States. A multivariable logistic regression model was conducted to identify key factors influencing expenditures.

**RESULTS:**

For patients aged 50 and older, the burden for all age groups showed a modest increase over the 6-year period. Annual medical expenditures were estimated to range from US$24.8 billion to US$39.2 billion from 2014 to 2019. The annual loss in productivity for patients was approximately US$1,200. The top 3 major components of medical costs were hospital inpatient stays, prescription medicines, and office-based visits. Medicare was the largest source of payments for survivors. In terms of drug consumption, genitourinary tract agents (57.0%) and antineoplastics (18.6%) were the main therapeutic drugs. High medical expenditures were positively associated with age (p=0.005), having private health insurance (p=0.016), more comorbidities, not currently smoking (p=0.001), and patient self-perception of fair/poor health status (p<0.001).

**CONCLUSIONS:**

From 2014 to 2019, the national real-world data of PC revealed that the disease burden in the United States continued to increase, which was partly related to patient characteristics.

## INTRODUCTION

Prostate cancer (PC) is the second most frequent cancer in men and the fifth leading cause of death worldwide [1]. In the United States, with an estimated 248,530 new cases of PC and 34,140 deaths in 2021 [2], it accounts for 36% of all men cancer cases and 13% of all men cancer-related deaths [3], and PC has a 5-year relative survival rate of 97.5% [4]. Compared with other cancers, its survival period is relatively long. In fact, more than 3.1 million men in the United States have been diagnosed with PC [5]. Survivors may impose a heavy burden on national healthcare expenditures as cumulative patient survival rates improve.

The Centers for Medicare & Medicaid Services (CMS) estimated that total national healthcare expenditures reached US$3.8 trillion in 2019, up from US$3.6 trillion in 2018 [6]. However, relatively few studies have been conducted on the PC burden at the national level in the United States in recent years. Brawley [7] demonstrated increasing trends in incidence, mortality, and survival rates from 1975 to 2007, and revealed risk factors such as age, family history, ethnicity, screening behavior, and obesity. The cost of cancer care is expected to rise substantially with advances in diagnostic technology and therapeutic drugs [8]. Therefore, it is necessary to re-evaluate the burden of PC in the United States.

Based on the updated data reported by the Global Burden of Disease (GBD) Study 2019, the disease burden of PC in 2014-2019 was evaluated. More importantly, the Medical Expenditure Panel Survey (MEPS) was also used to explore total medical expenditures, productivity loss, and major prescription medication frequency and expenditures. This comprehensive study also assessed the relationship between influencing factors and direct healthcare expenditures in PC patients, providing insights into the recent costs and treatment patterns of this disease, as well as the rational allocation of healthcare resources.

## MATERIALS AND METHODS

### Data source and study population

This study analyzed the epidemiological statistics, healthcare expenditures, and productivity loss of PC in the United States using the GBD 2019 and MEPS data from 2014 to 2019.

The GBD 2019 is the latest database of studies on the global burden of disease, providing tools for quantifying health loss from hundreds of diseases, injuries, and risk factors [9]. It is the most comprehensive worldwide observational epidemiological study to date [10], and it has consistently been used to assess the burden of diseases and injuries in different countries from various perspectives [11].

The MEPS is a set of large-scale surveys that provide nationally representative estimates of healthcare use, expenditure, sources of payment, and health insurance coverage for the United States civilian non-institutionalized population [12]. The MEPS collects data by interviewing households and individuals about specific health services, including how frequently they use those services, how much they cost, and how they are paid. Ultimately, 958 patients were identified using the International Classification of Diseases code, representing PC patients nationally from 2014 to 2019 (Supplementary Material 1). The data files in MEPS were all consolidated using unique personal identifiers to allow researchers to acquire information on personal healthcare spending and usage.

### Outcome measures

The burden of PC includes epidemiological statistics, healthcare expenditures, and productivity loss (Supplementary Material 2). Data on the number of deaths, morbidity, prevalence, and disability-adjusted life years (DALYs) of PC were available from an online query tool, the Global Health Data Exchange. DALYs are a measure of the overall disease burden, expressed as the cumulative number of years lost due to ill-health, disability, or early death, and serve as a composite measure of life quantity and quality in terms of time [13].

In the MEPS, the healthcare expenditures are the total direct healthcare costs in a year for each patient, including office-based visits, outpatient visits, emergency room visits, hospital inpatient care, prescription medicines, home healthcare, and other healthcare services. The payment sources include out-of-pocket, Medicare, Medicaid, private insurance, and other payment coverage. All costs over the 2014-2019 period were inflated to 2019 US dollars based on the consumer price index from the U.S. Bureau of Labor Statistics [14].

The expenditures and frequency of prescription medication for PC patients were described using the MEPS Prescribed Medicines File. All medications were identified by generic names. These medications were grouped into several categories, including antineoplastics, agents for comorbidities, analgesics, and so forth.

### Patient-level characteristics

Patient demographic variables were age, race, marital status, educational attainment, family income level, health insurance coverage, census tract, comorbidities, smoking status, and perceived health status. Age was categorized as 18-49 years, 50-64 years, 65-79 years, and ≥ 80 years, and race was categorized as White and other. Health insurance coverage was classified into private health insurance and non-private health insurance. Educational attainment was classified as (1) high school graduate or less or (2) at least some college. Marital status was defined as married or unmarried, with the unmarried category including those who reported being widowed, divorced, separated, and never married. The census region was divided into the Northeast, Midwest, South, and West. The income level was grouped into 3 categories: poor and near-poor, low and middle income, and high income. Comorbidities included hypertension, stroke, emphysema, high cholesterol, diabetes, arthritis, and asthma. Perceived health status was defined as “excellent/good” or “fair/poor.”

### Statistical analysis

Medical expenditures in the MEPS were right-skewed, so they were logarithmically transformed to an approximately normal distribution for parameter inspection. Then, a univariate regression model was used to calculate medical expenditure estimates in patients by factoring in differences in patient-level characteristics. Statistical comparisons were 2-sided, and statistical significance was defined as a p-value less than 0.05. Only predictors with a p-value less than 0.05 were retained in the multivariable logistic regression model, which was used to estimate the expenditure ratio for influencing factors associated with the medical expenditures of PC patients.

We evaluated the productivity loss costs by the probability of employment disability and the number of workdays missed due to PC. Logistic regression was used to estimate the probability of employment disability, while negative binomial regression was used to estimate the number of workdays missed. The humancapital method was used to calculate productivity loss by multiplying the probability of employment disability (number of workdays missed) by the average annual wage in 2019 (average daily wage in 2019) [15]. MEPS sampling weight variables were used to account for the complex survey design and calculate nationally representative estimates. The total national expenditures associated with PC were extrapolated by multiplying the estimated individual healthcare expenses by the MEPS sample weights. Analytic files were created using Stata version 16.0 (StataCorp., College Station, TX, USA) and analyses were performed in SPSS version 20.0 (IBM Corp., Armonk, NY, USA).

### Ethics statement

The data that support the findings of this study are open to public use and available from http://ghdx.healthdata.org/gbd-result-stool and https://meps.ahrq.gov/mepsweb/index.jsp for which may not be needed ethics approval.

## RESULTS

### Patient-level characteristics

Most PC patients were older than 65 years, non-Hispanic White, and with low and middle income or high income. Approximately 60% of PC patients were married. No significant imbalance was found in terms of educational level (below and above high school). The proportion of privately insured patients was almost identical to that of non-privately insured patients. Nearly 40% of patients came from the South of the United States. Hypertension, high cholesterol, and arthritis were the most common comorbidities in these patients. Nearly 90% of the patients were not current smokers and 70% felt good about their health (Table 1).

### Epidemiological statistics

**Figure 1.**
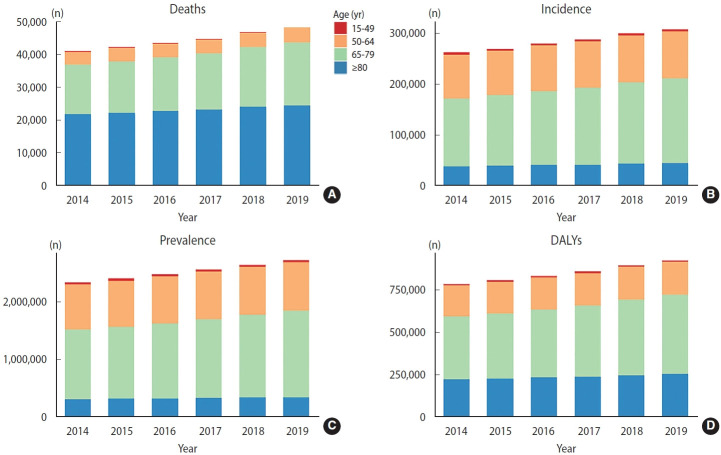
Prostate cancer cases (A) death, (B) incidence, (C) preva;emce. and (D) disability-adjusted life years (DALYs) and age composition in the United States, 2014-2019.

For patients over 50 years old, the mortality, morbidity, prevalence, and DALYs in the other age groups modestly increased over the 6-year study period (Figure 1). About 50% of deaths occurred in patients over 80 years old (Figure 1A), followed by those aged 65-79 years old (about 36% of deaths). Similarly, these 2 age subgroups comprised the largest cohort of existing PC patients in the United States (Figure 1C). The incidence of PC continued to increase in all age groups except for the 15-49 age subgroup (Figure 1B), with the most pronounced increase in the 65-year-old to 79-year-old group. The DALYs of patients over 50 years old increased year by year, with the highest growth rate in 65-79 age subgroup and the annual total DALYs up to 801,162 in 2019 (Figure 1D). The rates of 4 age-standardized indicators for PC in the United States all showed a continuous increase (Supplementary Material 3). The largest increase was found in 2018, which showed an increase of 4.6% (95% confidence interval [CI], 3.5 to 6.6) per 100,000 person-years in the age-standardized DALY rates.

### Healthcare expenditures and productivity loss

**Figure 2.**
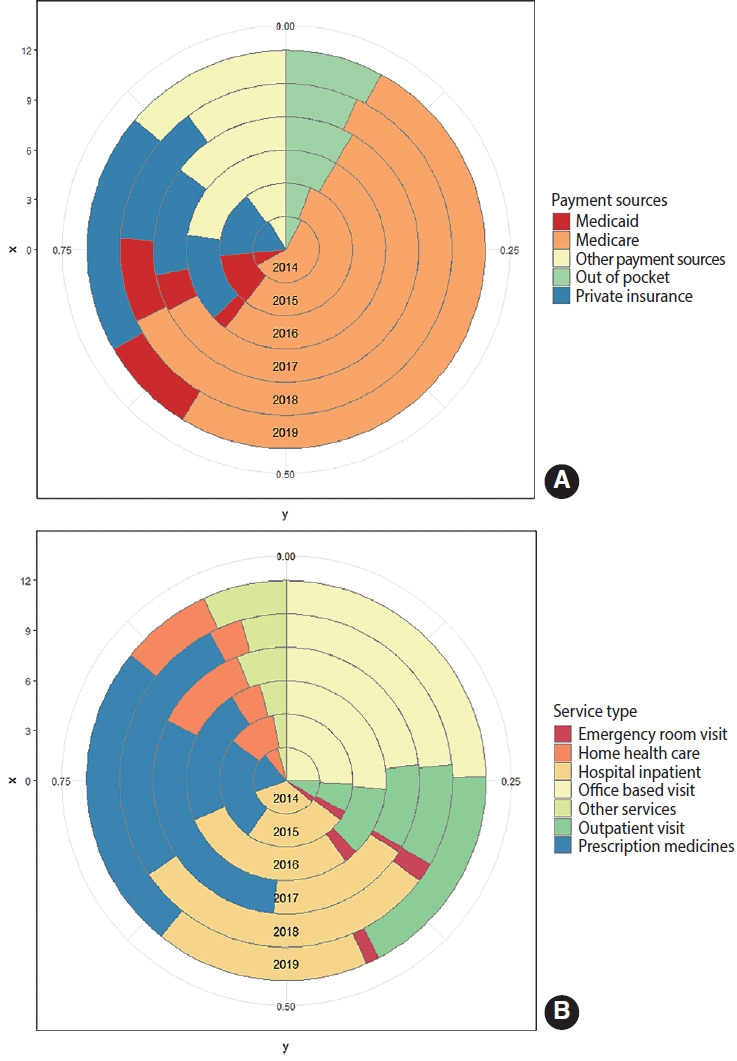
The proportions of medical expenditures for prostate cancer survivors in the United States, 2014-2019. (A) Categorized by source of payment. (B) Categorized by type of service.

The results for healthcare expenditures and productivity loss from 2014 to 2019 in PC survivors are displayed in Supplementary Material 4. The proportion of medical expenditures relative to total expenses ranged from 91.3% to 97.0% (Supplementary Material 5A). The national medical expenditures for PC ranged from US$24.8 billion to US$39.2 billion, with the highest in 2017 (Supplementary Material 5B). Figure 2 shows the proportions of medical expenditures by payment source and types of services. Medicare was the largest source of payment for PC survivors (Figure 2A). Office-based visits, hospital inpatient care, and prescription medicines were the top 3 sources of spending on healthcare services, together accounting for about 70% of total healthcare spending (Figure 2B).

**Figure 3.**
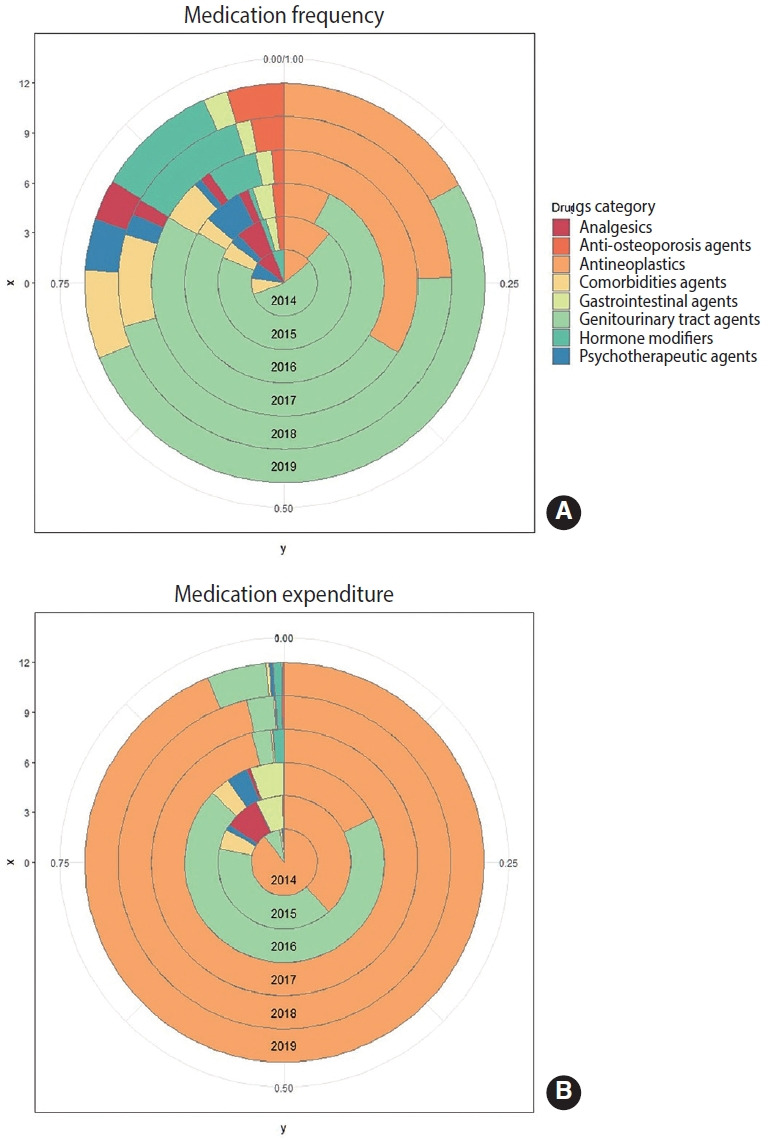
The proportions of main prescription medication use for prostate cancer survivors in the United States, 2014-2019. (A) The results of main prescription medication frequency. (B) The results of main prescription medication expenditure.

An analysis of the main prescription drug frequency and expenditures of PC patients showed that genitourinary tract agents (57.0%) and antineoplastics (18.6%) were the most frequently consumed treatment drugs for these patients, followed by hormone modifiers (6.4%) and agents for comorbidities (6.2%) (Supplementary Material 6). Notably, with the exception of 2015 and 2016, antineoplastics accounted for about 20% of medication frequency (Figure 3A), but approximately 80% or more of expenditures (Figure 3B) in most years.

Multivariate logistic regression models showed that high medical costs were associated with older age, married men, private health insurance, currently non-smoking, self-perceived fair or poor health status, and the presence of comorbid high cholesterol, arthritis, or diabetes. For PC survivors in the United States, older patients (> 80 years old) had 86% higher medical costs than younger (<50 years old) patients (p= 0.005). Patients with private insurance paid 41% more than those without private insurance (p = 0.016). Patients with excellent/good self-perceived health showed 67% lower healthcare spending than those with self-perceived fair/poor health (p< 0.001). Medical expenses for patients who were not current smokers were 54% higher than those for patients who presently smoked (p= 0.001). Moreover, PC patients with high cholesterol, arthritis, or diabetes had nearly 50% higher medical expenditures than those without comorbidities (Table 2).

## DISCUSSION

The CMS National Health Expenditure Data Project estimated that United States healthcare spending is expected to surge from US$3.6 trillion to US$6.0 trillion from 2018 to 2027, with increases in the cost of anticancer treatments being the primary cause [16]. With advances in diagnostic and treatment, cancer healthcare spending is likely to grow at the highest rate of all categories of healthcare spending. Understanding how medical expenditures vary by demographic characteristics, health insurance coverage, comorbidities, and census tracts is important for developing healthcare policies in targeted areas. The cost of cancer has become unaffordable in many countries, and in the United States, even patients with insurance are required to pay significant out-of-pocket costs.

Faced with such a dilemma, cancer survivors have been forced to go into debt or delay retirement, causing financial harm to patients. As one of the most common cancers and the second leading cause of cancer death among men in the United States, the burden of PC on the nation and individuals should not be ignored. Based on the latest GBD and MEPS data, this comprehensive study found that PC was associated with an enormous burden, and given the complexity of cancer management in these patients, it will challenge already strained healthcare systems in the United States. Our study advocates for considering the potential contributors to increased spending on PC, and clinicians, policymakers, and researchers are expected to reduce the substantial burden of PC, which will progressively affect survivors and their families and even national medical expenditures over the next decades.

Our results suggest that old age is a risk factor for PC [17]. However, early-onset PC may be associated with a substantially poorer prognosis and increased disease burden [18]. Our research suggests that the incidence of PC has been declining steadily from 2014 to 2019, demonstrating a positive trend in a way, but this cohort of PC cases still deserves more attention. A study reported that in 2012, patients diagnosed with early-onset PC accounted for approximately 10% of cases in the United States [19], which strengthened our finding of 6% in 2014 as indicative of a decreasing trend. Several studies have focused on the correlation between the age at PC diagnosis and disease burden. Bleyer et al. [20] found that since 1990, the global incidence of prostate cancer among 15-year-old to 40-year-old had increased at an average rate of 2% per year.

Younger PC patients were approximately 6 times more likely to have distant metastatic disease at diagnosis than older men. The overall 5-year relative survival rate was only 30% in those aged 15 to 24, 50% in those aged 20 to 29, 80% in those aged 25 to 34, and 95% in 100% in those in their 40s to 80s [20]. Meanwhile, young cancer patients face not only the medical costs associated with treatment but also the costs associated with lost productivity, specifically in relation to limited work activities, missed days of work, and reduced household productivity. Moreover, they are more likely to have difficulties obtaining and maintaining employment, leading to higher unemployment rates and heavier economic burden [21].

Our study estimated that the average annual medical spending attributed to PC was approximately US$30.0 billion from 2014 to 2019, with the highest in 2017 (US$39.2 billion) and the lowest in 2016 (US$24.8 billion). The extreme peak in 2017 should be interpreted with caution due to the sampling error in the database and the change in national health policy. The MEPS sampled the population to provide nationally representative estimates of healthcare utilization, and the unavoidable sampling errors may have led to unexplained variation in the results.

Furthermore, the Affordable Care Act (ACA), a comprehensive healthcare reform including a series of health-related provisions, was partly restricted in 2017. The ACA is a national health policy that went into effect in 2010 [22], and it expanded health insurance coverage for most Americans, lowered out-of-pocket expenditure for patients, and made prescription drugs cheaper for patients. The changes to the ACA may have influenced patient medication behavior, prompting a surge in spending in 2017. Annual medical spending dropped after 2017, but showed an upward trend after that.

A similar study compared over-adjusted annual healthcare expenditures and productivity loss between PC patients and individuals with no history of cancer [23]. In that study, nearly 60% of cancer survivors had at least 2 additional comorbidities. Cancer survivors with comorbidities incurred significantly healthcare expenditures than individuals without a history of cancer, which is consistent with our results. Previous studies have also linked comorbidities to higher expenditures [24]. Comorbidities can lead to an inability to work and prolonged bed rest, resulting in lost productivity and indirect increased spending.

This study has the following limitations. First, there is the potential for recall bias because MEPS data collection relied on self-reports or reports by family members. Some respondents were unable to accurately report health conditions. However, previous studies have shown that medical records and self-reported cancer history in MEPS were highly consistent [25].

Second, due to the cross-sectional nature of MEPS and the lack of important information on the timing of cancer diagnosis and survival, we were unable to stratify the sample by the time since diagnosis and estimate the cost of cancer care by stage [26]. In the future, the MEPS questionnaire design could be improved to capture this information. Third, patients with short survival may not have been able to complete 5 rounds of interviews during the survey period, resulting in insufficient data on cancer survivors with short survival, weakening the representativeness of the data. Population-based surveys may also lead to repeated surveys of selected patients in different years. However, based on the calculations in the study, only a very small fraction of patients was replicated. Moreover, each patient’s annual medical expenditures and individual health status vary from year to year, so this would not influence the general patterns of use and expenses for healthcare resources in the United States.

The burden of PC remained substantial in the United States in 2014-2019. Unlike patients aged below 50, the number of deaths, incidence, prevalence, and DALYs of PC have been increasing continuously in patients over 50 years old, especially those over 65. PC-related healthcare costs are high and persistent, and are related to socioeconomic factors such as age, private health insurance, comorbidities, smoking status, and patient self-perceived health status. Governments and medical institutions need to adjust medical strategies based on PC demographics and health insurance coverage, including precision screening, ongoing intervention programs, and healthcare policy reforms that can help reduce the burden of PC burden.

## DATA AVAILABILITY

The original contributions presented in the study are included in the article/Supplementary Materials. Further inquiries can be directed to the corresponding author.

## Figures and Tables

**Table 1.** Demographic characteristics of adults with prostate cancer, 2014-2019

Characteristics		2014	2015	2016	2017	2018	2019
Age (yr)							
	18-49	2 (1.4)	3 (1.8)	2 (1.3)	3 (1.9)	1 (0.6)	1 (0.6)
	50-64	25 (17.4)	30 (17.6)	23 (14.8)	28 (18.1)	31 (19.6)	34 (19.3)
	65-79	82 (56.9)	101 (59.4)	99 (63.9)	96 (61.9)	96 (60.8)	106 (60.2)
	≥80	35 (24.3)	36 (21.1)	31 (20.0)	28 (18.1)	30 (19.0)	35 (19.9)
Race							
	Non-Hispanic White	95 (66.0)	111 (65.3)	107 (69.0)	117 (75.5)	117 (74.1)	133 (75.6)
	Other	49 (34.0)	59 (34.7)	48 (31.0)	38 (24.5)	41 (25.9)	43 (24.4)
Marital status							
	Married	93 (64.6)	112 (65.9)	100 (64.5)	95 (61.3)	100 (63.3)	116 (65.9)
	Unmarried^1^	51 (35.4)	58 (34.1)	55 (35.5)	60 (38.7)	58 (36.7)	60 (34.1)
Educational attainment							
	≤High school	73 (50.7)	81 (47.4)	91 (58.7)	84 (54.2)	95 (60.1)	100 (56.8)
	≥Some college	71 (49.3)	87 (51.2)	63 (40.6)	71 (45.8)	63 (39.9)	76 (43.2)
Family income level							
	Poor and near poor	30 (20.8)	42 (24.7)	34 (12.9)	25 (16.1)	18 (11.4)	28 (15.9)
	Low and middle income	58 (40.3)	74 (43.5)	59 (38.1)	59 (38.1)	67 (42.4)	68 (38.6)
	High income	56 (38.9)	54 (31.8)	62 (40.0)	71 (45.8)	73 (46.2)	80 (45.5)
Health insurance							
	Private	69 (47.9)	78 (45.9)	65 (41.9)	84 (54.2)	76 (48.1)	85 (48.3)
	Non-private	75 (52.1)	92 (54.1)	90 (58.1)	71 (45.8)	82 (51.9)	91 (51.7)
Census region							
	Northeast	30 (20.8)	31 (18.2)	29 (18.7)	41 (26.5)	42 (26.6)	42 (23.9)
	Midwest	28 (19.4)	40 (23.5)	38 (24.5)	27 (17.4)	31 (19.6)	34 (19.3)
	South	53 (36.8)	60 (35.3)	58 (37.4)	61 (39.4)	55 (34.8)	61 (34.7)
	West	33 (22.9)	39 (22.9)	30 (19.4)	26 (16.8)	30 (19.0)	39 (22.2)
Comorbidities							
	Hypertension	116 (80.6)	136 (80.0)	116 (74.8)	106 (68.4)	104 (65.8)	118 (67.1)
	High cholesterol	114 (79.2)	111 (65.3)	96 (61.9)	97 (62.6)	105 (66.5)	108 (61.4)
	Arthritis	76 (52.8)	94 (55.3)	88 (56.8)	80 (51.6)	83 (52.5)	98 (55.7)
	Diabetes	31 (21.5)	39 (22.9)	40 (25.8)	43 (27.7)	46 (29.1)	38 (21.6)
	Stoke	20 (13.9)	27 (15.9)	23 (14.8)	20 (12.9)	17 (10.8)	31 (17.6)
	Asthma	18 (12.5)	19 (11.2)	13 (8.4)	10 (6.5)	16 (10.1)	16 (9.1)
	Emphysema	14 (9.7)	11 (6.5)	14 (9.0)	5 (3.2)	11 (7.0)	11 (6.3)
Current smoking							
	Yes	15 (10.4)	18 (10.6)	12 (7.7)	13 (8.4)	14 (8.9)	15 (8.5)
	No^2^	117 (81.3)	136 (80.0)	130 (83.8)	136 (87.7)	139 (88.0)	155 (88.1)
Perceived health status							
	Excellent/good	91 (63.2)	120 (70.6)	106 (68.4)	115 (74.2)	109 (69.0)	128 (72.7)
	Fair/poor	49 (34.0)	45 (26.5)	44 (28.4)	35 (22.6)	45 (28.5)	43 (24.4)

Values are presented as number (%).

1 Responses of “widowed,” “divorced,” “separated,” and “never married” were grouped in the “unmarried” category.

2 “No” for “current smoking” means that the respondents were non-smokers at the time of the interview, including ex-smokers and never-smokers.

**Table 2.** Summary of regression results for medical expenditures for patients with prostate cancer in the United States, 2014-2019^1^

Predictor variable	OR (95% CI)	p-value
Age (yr)		
18-49	0.14 (0.03, 0.55)	0.005
≥80	1.00 (reference)	
Health insurance		
Private	1.41 (1.07, 1.86)	0.016
Not private	1.00 (reference)	
Comorbidities		
High cholesterol		
Yes	1.44 (1.10, 1.89)	0.009
No	1.00 (reference)	
Arthritis		
Yes	1.45 (1.12, 1.87)	0.005
No	1.00 (reference)	
Diabetes		
Yes	1.50 (1.11, 2.02)	0.009
No	1.00 (reference)	
Current smoking		
Yes	0.46 (0.29, 0.72)	0.001
No^2^	1.00 (reference)	
Perceived health status		
Excellent/good	0.33 (0.25, 0.45)	<0.001
Fair/poor	1.00 (reference)	

OR, odds ratio; CI, confidence interval.

1 All statistical tests were 2-sided, and all p-values were derived from regression.

2 “No” for “current smoking” means that the respondents were non-smokers at the time of the interview, including ex-smokers and never-smokers.

## SUPPLEMENTARY MATERIALS

Supplementary Material 1. Flow diagram of included prostate cancer patients.

Supplementary Material 2 Diagram of outcome measured in the study.

Supplementary Material 3 The rate of age-standardized rates for prostate cancer cases in the United States from 2014-2019. (A) rates of deaths, (B) rates of incidence, (C) rates of prevalence and (D) rates of DALYs.

Supplementary Material 4 Total annual medical expenditure and productivity loss for individuals of prostate cancer in the MEPS, 2014-2019.

Supplementary Material 5 The sum of healthcare expenditures for prostate cancer survivors in the United States, 2014 – 2019. (A) the proportion of medical expenditure and productivity loss (B) estimated national medical expenditure.

Supplementary Material 6 Main prescription medication frequency and expenditure of prostate cancer patients by drug category, 2014-2019.
