# Supplementary material for: Disease burden of prostate cancer from 2014 to 2019 in the United States: estimation from the Global Burden of Disease Study 2019 and Medical Expenditure Panel Survey
Source: Epidemiol Health. 2023 Mar 21;45:e2023038. doi: 10.4178/epih.e2023038 (PMC10586921; doi:10.4178/epih.e2023038)
Supplement: Supplementary Material 1. — Flow diagram of included prostate cancer patients. [file epih-45-e2023038-Supplementary-1.docx]

**
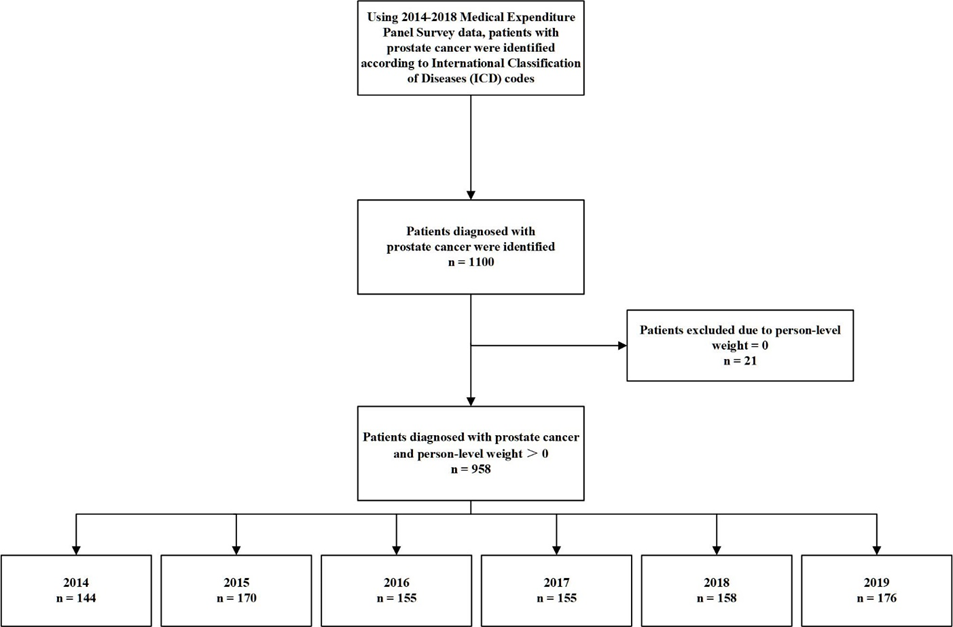
**

**Note:** Here in ICD code included the ICD-9 codes (code=185, before 2015) and ICD-10 codes (code=C61, after 2016) used to identify the patients.

**Supplementary Fig 1.** Flow diagram of included prostate cancer patients.
