# Supplementary figures and images for: Disease burden of prostate cancer from 2014 to 2019 in the United States: estimation from the Global Burden of Disease Study 2019 and Medical Expenditure Panel Survey
Source: Epidemiol Health. 2023 Mar 21;45:e2023038. doi: 10.4178/epih.e2023038 (PMC10586921; doi:10.4178/epih.e2023038)

**
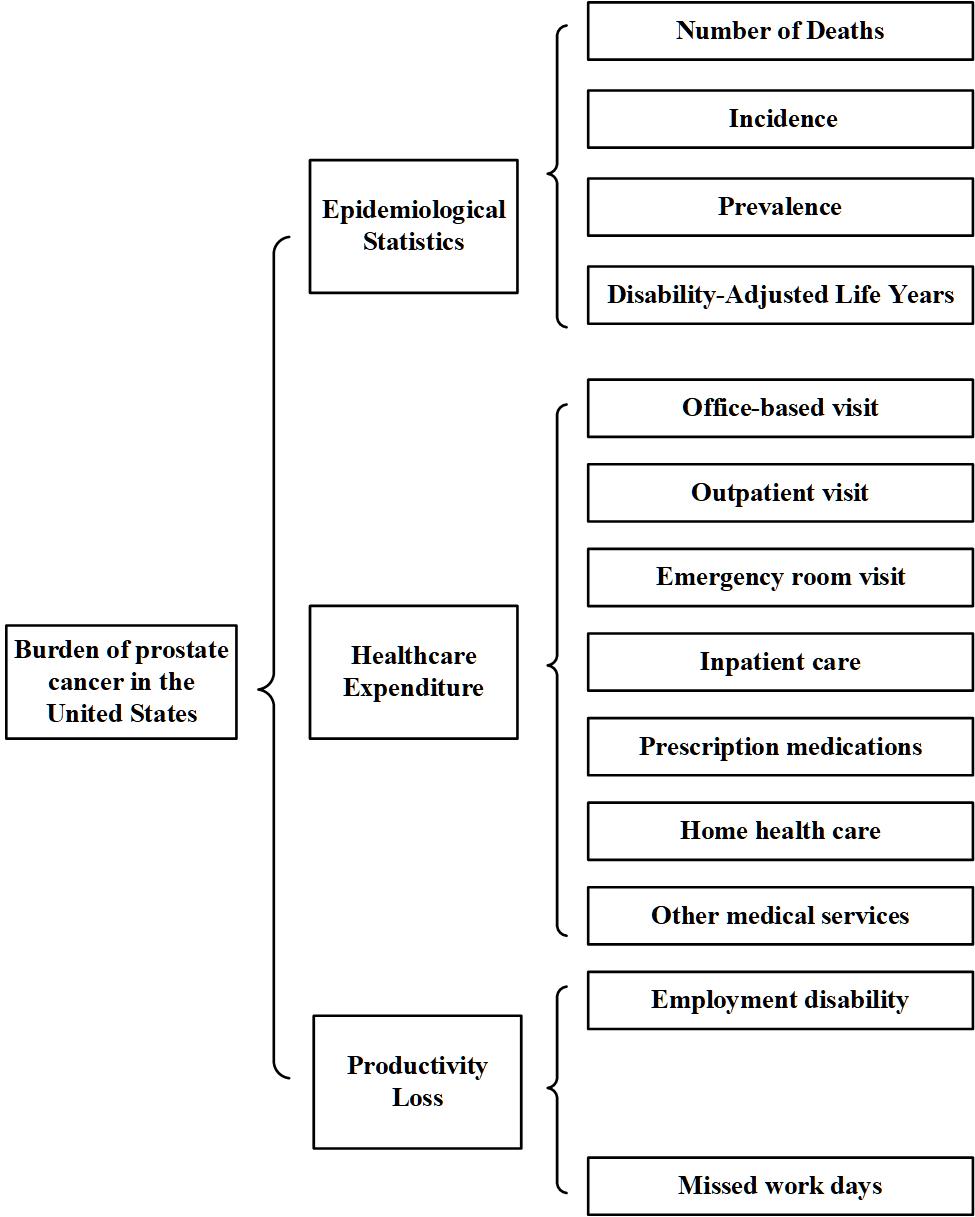
**

**Supplementary Fig 2.** Diagram of outcome measured in the study.

Supplement: Supplementary Material 2 — Diagram of outcome measured in the study. [file epih-45-e2023038-Supplementary-2.docx]
