# Supplementary material for: Disease burden of prostate cancer from 2014 to 2019 in the United States: estimation from the Global Burden of Disease Study 2019 and Medical Expenditure Panel Survey
Source: Epidemiol Health. 2023 Mar 21;45:e2023038. doi: 10.4178/epih.e2023038 (PMC10586921; doi:10.4178/epih.e2023038)
Supplement: Supplementary Material 3 — The rate of age-standardized rates for prostate cancer cases in the United States from 2014-2019. (A) rates of deaths, (B) rates of incidence, (C) rates of prevalence and (D) rates of DALYs. [file epih-45-e2023038-Supplementary-3.docx]

**
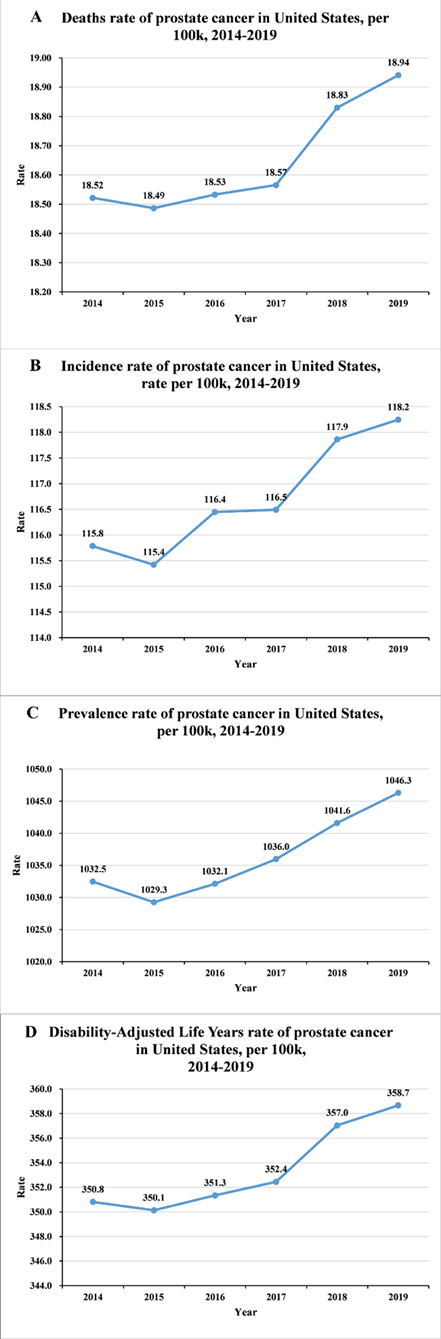
**

**Supplementary Fig 3.** The rate of age-standardized rates for prostate cancer cases in the United States from 2014-2019. (A) rates of deaths, (B) rates of incidence, (C) rates of prevalence and (D) rates of DALYs.
