# Supplementary material for: Disease burden of prostate cancer from 2014 to 2019 in the United States: estimation from the Global Burden of Disease Study 2019 and Medical Expenditure Panel Survey
Source: Epidemiol Health. 2023 Mar 21;45:e2023038. doi: 10.4178/epih.e2023038 (PMC10586921; doi:10.4178/epih.e2023038)
Supplement: Supplementary Material 4 — Total annual medical expenditure and productivity loss for individuals of prostate cancer in the MEPS, 2014-2019. [file epih-45-e2023038-Supplementary-4.docx]

**Supplementary Table 1.** Total annual medical expenditure and productivity loss for individuals of prostate cancer in the MEPS, 2014-2019.

| **Burden measurement** | **2014** | | **2015** | | **2016** | | **2017** | | **2018** | | **2019** | |
| --- | --- | --- | --- | --- | --- | --- | --- | --- | --- | --- | --- | --- |
|  | **Mean** | **95%CI** | **Mean** | **95%CI** | **Mean** | **95%CI** | **Mean** | **95%CI** | **Mean** | **95%CI** | **Mean** | **95%CI** |
| **Medical expenditure** | **18374** | **(14865, 21884)** | **15494** | **(11663, 19326)** | **14296** | **(11363, 17228)** | **21170** | **(16247, 26092)** | **26169** | **(19451, 32887)** | **19243** | **(15627, 22859)** |
| **Source of payment** |  |  |  |  |  |  |  |  |  |  |  |  |
| Medicare | 10899 | (7834, 13964) | 8421 | (5818, 11025) | 7498 | (5518, 9477) | 12503 | (8482, 16525) | 15914 | (10432, 21395) | 9767 | (7339, 12197) |
| Private health insurance | 3212 | (2045, 4380) | 2557 | (1445, 3669) | 2066 | (667, 3465) | 2828 | (1597, 4060) | 3628 | (2044, 5212) | 3776 | (1829, 5723) |
| Out-of-pocket | 1387 | (922, 1851) | 935 | (734, 1137) | 1196 | (851, 1540) | 1763 | (1335, 2191) | 1849 | (1329, 2369) | 1523 | (1185, 1861) |
| Medicaid | 1263 | (361, 2165) | 2021 | (-98, 4140) | 363 | (137, 590) | 961 | (-52, 1974) | 2148 | (-957, 5254) | 1536 | (323, 2750) |
| Other sources† | 1612 | (892, 2333) | 1559 | (745, 2373) | 3262 | (1459, 5066) | 3114 | (1464, 4763) | 2630 | (1500, 3762) | 2640 | (1250, 4030) |
| **Type of services** |  |  |  |  |  |  |  |  |  |  |  |  |
| Hospital Inpatient | 6104 | (3852, 8356) | 3829 | (2308, 5350) | 4115 | (2369, 5861) | 3695 | (1804, 5586) | 7905 | (3016, 12795) | 3330 | (1894, 4767) |
| Office-based Visit | 4636 | (3493, 5778) | 4009 | (2780, 5237) | 3777 | (2572, 4983) | 4884 | (3596, 6174) | 6143 | (3754, 8534) | 4752 | (3780, 5724) |
| Prescription Medicines | 3607 | (2273, 4942) | 3943 | (2249, 5637) | 3161 | (2003, 4320) | 6551 | (3918, 9184) | 7053 | (4013, 10092) | 4815 | (2818, 6813) |
| Outpatient Visit | 1504 | (867, 2141) | 1047 | (528, 1566) | 1600 | (677, 2524) | 2164 | (881, 3447) | 2582 | (1558, 3606) | 3392 | (1550, 5233) |
| Home Health Care | 1288 | (447, 2128) | 1801 | (-248, 3850) | 701 | (259, 1142) | 2447 | (367, 4527) | 834 | (293, 1376) | 1437 | (379, 2495) |
| Emergency Room Visit | 496 | (170, 821) | 356 | (184, 527) | 316 | (157, 476) | 154 | (80, 229) | 503 | (25, 982) | 218 | (100, 336) |
| Other Services‡ | 740 | (500, 981) | 509 | (362, 656) | 623 | (442, 805) | 1273 | (864, 1681) | 1148 | (837, 1458) | 1299 | (945, 1654) |
| **Productivity loss** | **1524** | **(1134, 1916)** | **1478** | **(1171, 1809)** | **1172** | **(897, 1518)** | **1279** | **(1004, 1555)** | **1211** | **(951, 1471)** | **1386** | **(1127,1701)** |
| **Employment disability (%)** | 2.1 | (1.6, 2.6) | 2.1 | (1.7, 2.6) | 1.5 | (1.1, 2.0) | 1.7 | (1.3, 2.1) | 1.6 | (1.2, 2.0) | 1.9 | (1.5, 2.4) |
| Per capita productivity loss§ | 1123 | (856, 1391) | 1123 | (909, 1391) | 802 | (588, 1070) | 909 | (695, 1123) | 856 | (642, 1070) | 1016 | (802, 1284) |
| **Missed work days** | 2.6 | (1.8, 3.4) | 2.3 | (1.7, 2.7) | 2.4 | (2.0, 2.9) | 2.4 | (2.0, 2.8) | 2.3 | (2.0, 2.6) | 2.4 | (2.1, 2.7) |
| Per capita productivity loss¶ | 401 | (278, 525) | 355 | (262, 417) | 370 | (309, 448) | 370 | (309, 432) | 355 | (309, 401) | 370 | (324, 417) |
| **Annual burden** | **19898** | **(15999, 23800)** | **16972** | **(12834, 21135)** | **15468** | **(12260, 18746)** | **22449** | **(17251, 27647)** | **27380** | **(20405, 34358)** | **20629** | **(16754, 24560)** |

†Other source of insurance payment included Veterans Administration, and the Civilian Health and Medical Program of the Department of Veterans Affairs.

‡ Other healthcare services included dental and vision services, other medical equipment and services.

§ A logistic regression model was utilized. The estimated productivity loss was obtained by multiplying the probability of employment disability by the 2019 average annual wage ($53,490).

¶ A negative binomial regression model was utilized. The estimated productivity loss was obtained by multiplying the number of missed work days by the 2019 average daily wage ($25.72/hour * 6 hours=$154.32/day).
