# Supplementary material for: Disease burden of prostate cancer from 2014 to 2019 in the United States: estimation from the Global Burden of Disease Study 2019 and Medical Expenditure Panel Survey
Source: Epidemiol Health. 2023 Mar 21;45:e2023038. doi: 10.4178/epih.e2023038 (PMC10586921; doi:10.4178/epih.e2023038)
Supplement: Supplementary Material 5 — The sum of healthcare expenditures for prostate cancer survivors in the United States, 2014 – 2019. (A) the proportion of medical expenditure and productivity loss (B) estimated national medical expenditure. [file epih-45-e2023038-Supplementary-5.docx]

**
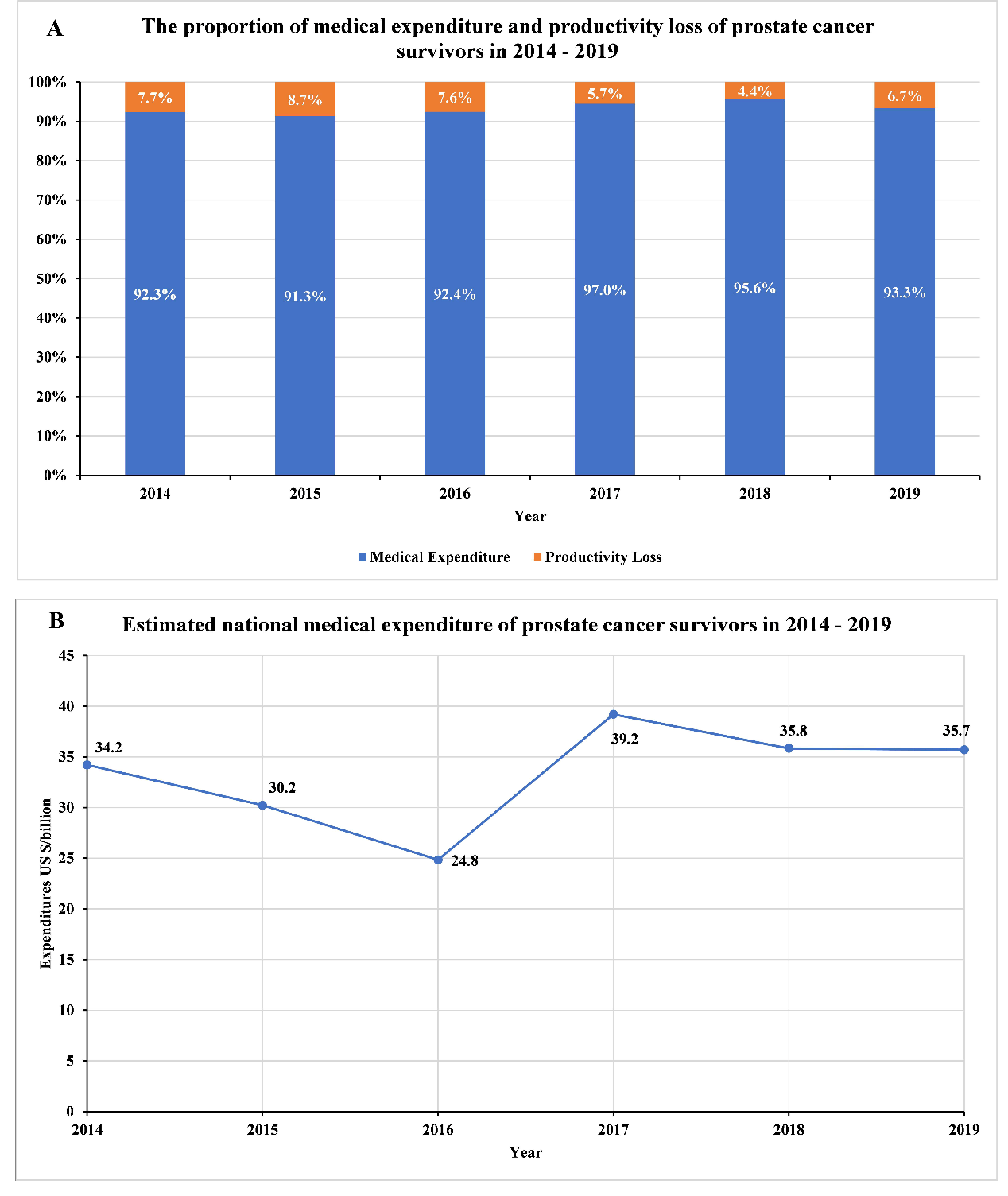
**

**Supplementary Fig 4.** The sum of healthcare expenditures for prostate cancer survivors in the United States, 2014 – 2019. (A) the proportion of medical expenditure and productivity loss (B) estimated national medical expenditure.
