# Supplementary material for: Disease burden of prostate cancer from 2014 to 2019 in the United States: estimation from the Global Burden of Disease Study 2019 and Medical Expenditure Panel Survey
Source: Epidemiol Health. 2023 Mar 21;45:e2023038. doi: 10.4178/epih.e2023038 (PMC10586921; doi:10.4178/epih.e2023038)
Supplement: Supplementary Material 6 — Main prescription medication frequency and expenditure of prostate cancer patients by drug category, 2014-2019. [file epih-45-e2023038-Supplementary-6.docx]

**Supplementary Table 2.** Main prescription medication frequency and expenditure of prostate cancer patients by drug category, 2014-2019.

| **Year** | **Drugs category** | **Sum ($)** | **Rx No. (%)** | **Mean cost (95%CI) ($)** |
| --- | --- | --- | --- | --- |
| **2014** | Antineoplastics | 145358.8 | 42 (14.0) | 3460.9 (2220.6, 4701.2) |
|  | Genitourinary Tract Agents | 13000.7 | 166 (55.3) | 78.3 (59.1, 97.5) |
|  | Comorbidities Agents | 1943.8 | 24 (8.0) | 81.0 (50.8, 111.2) |
|  | Psychotherapeutic Agents | 1133.6 | 24 (8.0) | 47.2 (-5.5, 99.9) |
|  | Analgesics | 701.9 | 26 (8.7) | 27.0 (14.8, 39.2) |
|  | Hormone Modifiers | 215.5 | 18 (6.0) | 12.0 (10.2, 13.7) |
|  | Gastrointestinal Agents | - | - | - |
|  | Anti-Osteoporosis Agents | - | - | - |
| **2015** | Genitourinary Tract Agents | 12472.8 | 199 (69.3) | 62.7 (53.6, 71.8) |
|  | Antineoplastics | 11962.1 | 34 (11.8) | 351.8 (242.4, 461.3) |
|  | Analgesics | 2644.2 | 18 (6.3) | 146.9 (56.1, 237.7) |
|  | Gastrointestinal Agents | 1991.2 | 7 (2.4) | 284.5 (9.3, 559.7) |
|  | Comorbidities Agents | 1490.0 | 12 (4.2) | 124.2 (-4.0, 252.3) |
|  | Psychotherapeutic Agents | 456.0 | 7 (2.4) | 65.1 (30.2, 100.1) |
|  | Anti-Osteoporosis Agents | 147.8 | 6 (2.1) | 24.6 (12.1, 37.2) |
|  | Hormone Modifiers | 36.9 | 4 (1.4) | 9.2 (0.3, 18.1) |
| **2016** | Genitourinary Tract Agents | 18574.0 | 191 (77.3) | 97.2 (80.5, 114.0) |
|  | Antineoplastics | 4689.5 | 19 (7.7) | 246.8 (-164.8, 658.5) |
|  | Gastrointestinal Agents | 1440.3 | 8 (3.2) | 180.0 (58.0, 302.0) |
|  | Psychotherapeutic Agents | 929.1 | 16 (6.5) | 58.1 (14.4, 101.7) |
|  | Comorbidities Agents | 864.4 | 7 (2.8) | 123.5 (-131.9, 378.8) |
|  | Analgesics | 127.4 | 4 (1.6) | 31.9 (2.4, 61.3) |
|  | Anti-Osteoporosis Agents | 45.7 | 5 (2.0) | 9.1 (8.9, 9.3) |
|  | Hormone Modifiers | 14.9 | 2 (0.8) | 7.5 (7.5, 7.5) |
| **2017** | Antineoplastics | 431641.4 | 106 (33.8) | 4072.1 (3290.3, 4853.9) |
|  | Genitourinary Tract Agents | 10296.4 | 155 (49.4) | 66.4 (51.9, 80.9) |
|  | Hormone Modifiers | 6126.7 | 19 (6.1) | 322.5 (72.5, 572.4) |
|  | Comorbidities Agents | 685.4 | 16 (5.1) | 42.8 (12.9, 72.7) |
|  | Analgesics | 325.9 | 4 (1.3) | 81.5 (-88.7, 251.7) |
|  | Gastrointestinal Agents | 113.2 | 6 (1.9) | 18.9 (14.9, 22.9) |
|  | Psychotherapeutic Agents | 110.6 | 3 (1.0) | 36.9 (36.9, 36.9) |
|  | Anti-Osteoporosis Agents | 39.0 | 5 (1.6) | 7.8 (-6.9, 22.5) |
| **2018** | Antineoplastics | 303533.6 | 82 (24.5) | 3701.6 (2682.2, 4721.0) |
|  | Genitourinary Tract Agents | 8244.4 | 155 (46.3) | 53.2 (41.3, 65.0) |
|  | Hormone Modifiers | 1790.9 | 40 (11.9) | 44.8 (36.5, 53.1) |
|  | Anti-Osteoporosis Agents | 772.7 | 11 (3.3) | 70.2 (-71.0, 211.5) |
|  | Comorbidities Agents | 351.2 | 30 (9.0) | 11.7 (8.7, 14.7) |
|  | Psychotherapeutic Agents | 209.9 | 7 (2.1) | 30.0 (27.3, 32.7) |
|  | Analgesics | 96.4 | 5 (1.5) | 19.4 (15.8, 23.0) |
|  | Gastrointestinal Agents | 61.1 | 5 (1.5) | 12.2 (12.2, 12.2) |
| **2019** | Antineoplastics | 198221.0 | 61 (16.8) | 3249.5 (2110.3, 4388.7) |
|  | Genitourinary Tract Agents | 10231.7 | 189 (52.1) | 54.1 (44.6, 63.7) |
|  | Hormone Modifiers | 1362.5 | 36 (9.9) | 37.8 (19.1, 56.6) |
|  | Psychotherapeutic Agents | 595.9 | 15 (4.1) | 39.7 (29.6, 49.9) |
|  | Comorbidities Agents | 474.8 | 26 (7.2) | 18.3 (11.8, 24.7) |
|  | Anti-Osteoporosis Agents | 317.6 | 17 (4.7) | 18.7 (15.0, 22.4) |
|  | Gastrointestinal Agents | 213.0 | 7 (1.9) | 29.4 (7.1, 51.7) |
|  | Analgesics | 125.6 | 12 (3.3) | 9.5 (9.2, 9.7) |
| **Total** | Antineoplastics | 1095406.6 | 344 (18.6) | 3184.3 (2745.5, 3623.1) |
|  | Genitourinary Tract Agents | 72819.9 | 1055 (57.0) | 69.0 (63.4, 74.7) |
|  | Hormone Modifiers | 9547.4 | 119 (6.4) | 80.2 (38.2, 122.2) |
|  | Comorbidities Agents | 5809.7 | 115 (6.2) | 50.5 (31.0, 70.1) |
|  | Analgesics | 4009.9 | 69 (3.7) | 58.1 (31.5, 84.7) |
|  | Gastrointestinal Agents | 3811.9 | 33 (1.8) | 115.5 (50.4, 180.6) |
|  | Psychotherapeutic Agents | 3435.1 | 72 (3.9) | 47.7 (28.4, 67.0) |
|  | Anti-Osteoporosis Agents | 1322.6 | 44 (2.4) | 30.1 (-1.7, 61.8) |

**CI:** Confidence Interval.

All monetary values were adjusted to 2018 USD using the consumer price index for medical care.

**Note:**

Antineoplastics include antineoplastic hormones, Letrozole, Anastrozole, Tamoxifen.

Genitourinary tract agents include prostatic hyperplasia agents (Tamsulosin, Finasteride, Terazosin, etc.), urinary antispasmodics agents (Oxybutynin, Solifenacin, Tolterodine, and Mirabegron), and sexual dysfunction agents (Sildenafil and Tadalafil).

Comorbidities Agents include cardiovascular agents (Losartan, Amlodipine Atorvastatin, etc.) and antidiabetic agents (Metformin, Glimepiride, etc.)

Analgesics include Ibuprofen, Oxycodone, Acetaminophen-Hydrocodone, Morphine, Tramadol, Gabapentin, etc.

Gastrointestinal agents include laxatives (Docusate), proton pump inhibitors (Omeprazole, Pantoprazole, etc.) and antiemetic agents (Prochlorperazine and Doxylamine-Pyridoxine)

Psychotherapeutic Agents include selective serotonin reuptake inhibitors (Paroxetine, Escitalopram, etc.), serotonin-norepinephrine reuptake inhibitors (Duloxetine, Venlafaxine, etc.) and benzodiazepine (Diazepam, Lorazepam, etc.)

Hormone Modifiers include androgens and anabolic steroids (Testosterone), glucocorticoids (Prednisone, Dexamethasone) and Thyroid drugs (Levothyroxine).

Anti-Osteoporosis Agents include bone resorption inhibitors (Alendronate), Vitamin D Agents (Ergocalciferol and Calcitriol) and Calcium Agents (Calcium Carbonate and Calcium-vitamin D).
